# The etiology and prevention of feeding intolerance paralytic ileus – revisiting an old concept

**DOI:** 10.1186/1750-1164-3-3

**Published:** 2009-04-17

**Authors:** Gerald Moss

**Affiliations:** 1 Rensselaer Polytechnic Institute, Biomedical Engineering Department, Troy, New York, USA

## Abstract

Gastro-intestinal (G-I) motility is impaired ("paralytic ileus") after abdominal surgery. Premature feeding attempts delay recovery by inducing "feeding intolerance," especially abdominal distention that compromises respiration. Controlled studies (e.g., from Sloan-Kettering Memorial Hospital) have lead to recommendations that patients not be fed soon after major abdominal surgery to avoid this complication.

We postulate that when total fluid inflow of feedings, digestive secretions, and swallowed air outstrip peristaltic outflow from the feeding site, fluid accumulates. This localized stagnation triggers G-I vagal reflexes that further slow the already sluggish gut, leading to generalized abdominal distention. Similarly, vagal cardiovascular reflexes in susceptible subjects could account for the 1:1,000 incidence of unexplained bowel necrosis reported with enteral feeding.

We re-evaluated our data, which supports this postulated mechanism for the induction of "feeding intolerance." We had focused our efforts on postoperative enteral nutrition, with the largest reported series of immediate feeding of at least 100 kcal/hour after major surgery. We found that this complication can be avoided consistently by monitoring inflow versus peristaltic outflow, immediately removing any potential excess from the feeding site.

We fed intraduodenally immediately following "open" surgery for 31 colectomy and 160 consecutive cholecystectomy patients. The duodenum was aspirated simultaneously just proximal to the feeding site, efficiently removing all swallowed air and excess feedings. To salvage digestive secretions, the degassed aspirate was re-introduced manually (and later automatically) via a separate feeding channel.

Hourly assays were performed for nitrogen balance, serum amino acids, and for the presence of removed feedings in the aspirate. The colectomy patients had X-ray motility studies initiated 5 – 17 hours after surgery.

Clinically normal motility and absorption resumed within two hours. Fed BaSO_4 _traversed secure anastomoses, to exit in bowel movements within 24–48 hours of colectomy. All patients were in positive protein balance within 2 – 24 hours, with elevated serum amino acids levels and without adverse G-I effects.

Limiting inflow to match peristaltic outflow from the feeding site consistently prevented "feeding intolerance." These patients received immediate full enteral nutrition, with the most rapid resolution of postoperative paralytic ileus, to date.

## Introduction

We have the largest reported series of postoperative patients immediately fed ≥ 100 kcal/hr without encountering "feeding intolerance" We re-examined our data, reaching a conclusion that had escaped us at the time. Our patients had been spared the risk of overfeeding because we had avoided the trigger, which was localized distention at the enteral feeding site.

Our attention had been focused primarily on efficiently intercepting and removing swallowed air while feeding. We aspirated the proximal duodenum, where the fluid path was narrowed, efficiently removing that gas. Fortuitously, nutrition was introduced into the slightly more distal duodenum. Total inflow that exceeded the impaired peristaltic duodenal outflow refluxed freely within this short, relatively flaccid segment. This refluxing excess also was promptly removed via the more proximal duodenal aspiration orifices. The patients would not have fared as well had we fed into the jejunum.

We now postulate that when peristaltic outflow from the feeding site is exceeded by total inflow (feedings superimposed on digestive juices plus swallowed saliva and air), fluid accumulates at this proximal location. The vagally mediated reflex response is slowing of the already sluggish entire gut, leading to a "downhill spiral" of generalized abdominal distention, nausea, and malaise. Respiratory embarrassment develops secondarily. Unexplained bowel necrosis occurs after jejunal feeding, with a reported incidence of 1:1,000. This, too, might similarly follow vagal vascular reflexes in susceptible subjects [1].

Miedema, et al., at the University of Missouri, prospectively monitored jejunal pressure via the feeding catheter. Postoperative patients that developed "feeding intolerance" did demonstrate elevated feeding site pressure. However, the increased pressure developed too late to guide timely intervention [2]. Monitoring of volume, per se, apparently could be more useful.

Why do we want a safer means to feed early and aggressively? Postoperative metabolism resembles that of a growing child. Net protein synthesis is permissible and achievable, but only if adequate nutrition is made available. Trauma produces a hypermetabolic state. Increased energy output usually becomes the determinant of protein anabolism, and protein catabolism has been the rule. However, even net protein synthesis is possible. The major factor limiting adequate enteral nutrition at these times is paralytic ileus, an avoidable G-I complication [3].

No conventionally fed patient fully meets his increased nutritional needs for a variable period after major abdominal operation or comparable trauma. The potentially protective responses (e.g., heightened immune competence and accelerated wound healing) are blunted, limited by the patient's reduced gut function. Everyone's goal is to shorten this vulnerable, uncomfortable, and hospital dependent period.

Applying the latest "fast-track" techniques has progressively reduced the duration and severity of postoperative G-I dysfunction. Patients begin eating, reach nutritional goals, and are now discharged sooner, but they still endure days of documented negative protein balances after major abdominal resective surgery. We describe a regimen for achieving the earliest resolution of paralytic ileus and positive protein balances, to date.

## Materials and methods

The subjects were the author's 31 colectomy and 160 consecutive cholecystectomy patients (1962 – 1988), prior to the introduction of laparoscopy into general surgery. All cholecystectomy and nine of the earliest colectomy patients had a Moss^® ^naso-duodenal feeding-decompression device inserted (*Moss Tubes, Inc., West Sand Lake, NY). Subsequent bowel resection patients had the gastrostomy version of this feeding-decompression device placed. An elemental diet (Vivonex, Norwich-Eaton Pharmaceuticals, Norwich, NY) (now Nestle HealthCare Nutrition, Minnetonka, MN) was initiated at 100 – 300 kcal/hour within minutes of surgery.

The duodenal aspirate was tested hourly (Clinatest^® ^Tablets, Ames Co., Billerica, MA) for "reducing sugars," as a marker for the removed feeding solution. The "degassed" aspirate was filtered and returned manually via the feeding channel. Hourly nitrogen balances and serum amino acids were determined.

The nasal tube was removed the morning following surgery. The cholecystectomy patients were discharged after a bowel movement and tolerating a general diet. The colectomy patients transitioned to oral elemental diet and had BaSO_4 _X-ray motility study over the next 24 hours. They resumed a general diet and met the same discharge criteria.

The later colectomy patients, with placement of a gastrostomy version of the feeding-decompression device, had BaSO_4 _introduced with their feedings 4–6 hours following bowel resection and re-anastomosis. Motility was studied by X-ray for the duration of tube feeding.

## Results

The aspirate became Clinatest^® ^negative (reducing sugars absent) during the first or second postoperative hour. The total feedings were propelled forward thereafter by peristalsis. A maximum of 200 ml/day of refed gastroduodenal aspirate continued to reflux and was discarded. No patient developed abdominal distention or other signs of paralytic ileus or "feeding intolerance."

All patients had achieved positive nitrogen balance within 2–26 hours postoperative (Table 1). Similarly, serum branched chain amino acids rose above basal levels by 2–12 hours following surgery.

**Table 1:** Postoperative Protein Balances

		**Days**	**Average Daily Protein**			**Hours to**
**Operation**	**Age**	**Fed**	**Catabolized**	**Fed**	**Balance**	**(+) Balance**
1. Abdomino-perineal Resection	79	2	88	163	+ 75	15
2. Abdomino-perineal Resection	83	4	93	149	+ 56	4
3. Abdomino-perineal Resection	29	2	35	41	+ 6	24
4. Abdomino-perineal Resection	17	2	101	118	+ 17	8
5. Sigmoid Resection	60	2	104	122	+ 18	26
6. Sigmoid Resection	69	2	78	100	+ 22	7
7. Sigmoid Resection	76	2	42	77	+ 35	6
8. Sigmoid Resection	55	2	43	133	+ 90	22
9. Sigmoid & Bladder Resection	45	2	85	86	+ 1	18
10. Vagotomy	41	1	88	100	+ 17	7
11. Vagotomy	64	2	104	154	+ 50	6
12. Vagotomy & Pyloroplasty	70	4	68	150	+ 82	24
13. Vagotomy & Pyloroplasty	45	2	84	100	+ 16	18
14. Vagotomy & Pyloroplasty	60	2	85	136	+ 51	6
15. Vagotomy & Pyloroplasty	57	2	106	189	+ 83	6
16. V & P + Gastrectomy	38	2	31	85	+ 54	20
17. V & P + Gastrectomy + G.B.	60	2	98	104	+ 6	10
18. Hysterectomy	45	2	51	74	+ 23	6
19. Cholecystectomy	49	2	65	72	+ 7	24
20. Cholecystectomy	48	1	49	200	+151	2
21. Cholecystectomy	38	2	82	86	+ 4	17
22. Ileostomy Revision	32	1	38	140	+102	7
23. Ovarian Cystectomy	56	2	45	99	+ 54	23
24. Colostomy Closure	62	1	54	190	+136	4

(Data presented at the 1963 Surgical Forum – American College of Surgery [6])

**Figure 1:**
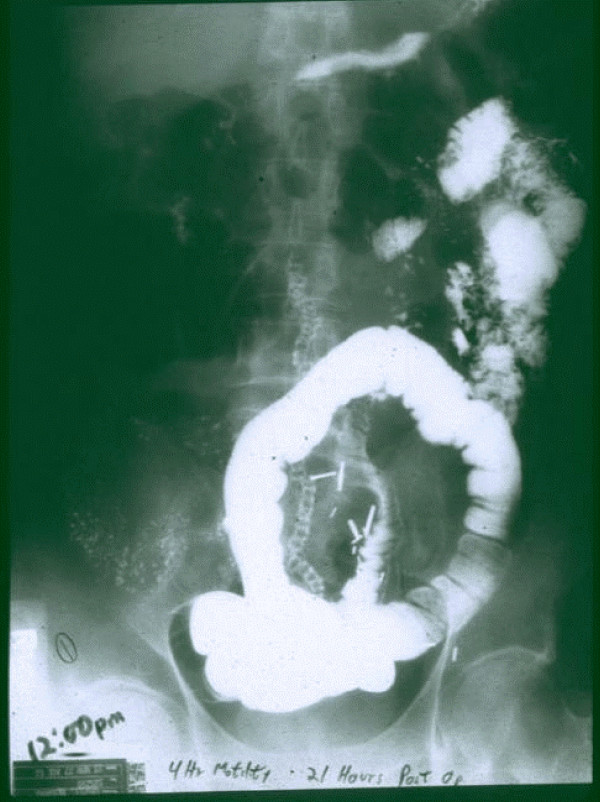
**66 y.o. male had sigmoid resection for carcinoma @ 3:00 pm**. He was fed immediately for 17 hours via a double lumen, nasoduodenal catheter @ 100 kcal/hr. At 8:00 am the tube was removed, and BaSO4 swallowed. Serial X-rays indicate normal motility. 12:00 noon – 4 hour motility study, 21 hours after surgery.

**Figure 2:**
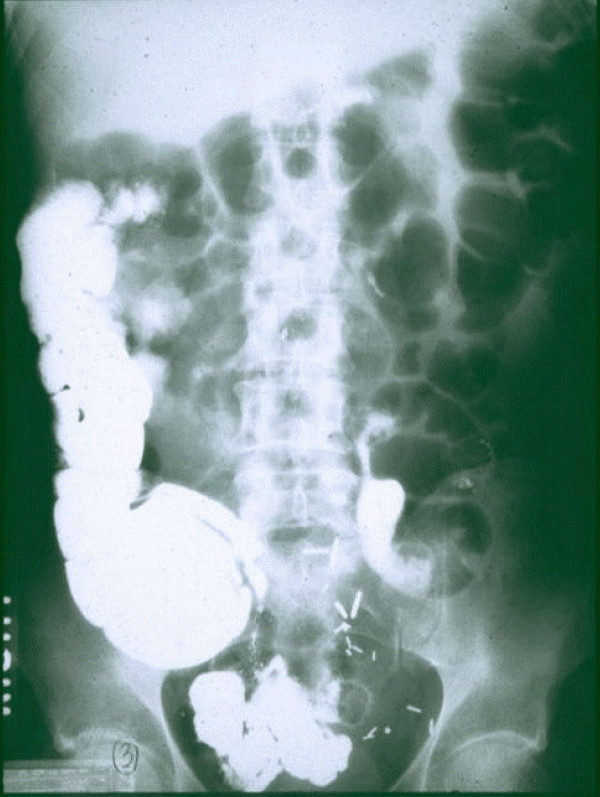
**Normal motility**. Contrast has reached the transverse colon. The more distal anastomosis is outlined by ring of stainless steel staples. 8:00 pm – 12 hour motility, 29 hours after sigmoid resection.

**Figure 3:**
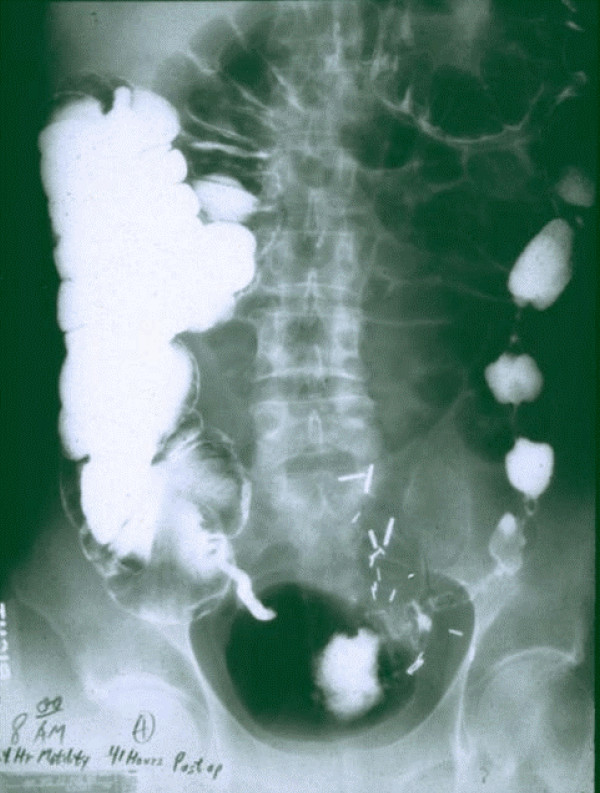
**BaSO4 has traversed the secure and patent stapled anastomosis, to outline the rectum**. The patient ate his Thanksgiving meal, and was discharged after a bowel movement within 48 hours of surgery. The X-ray shows a normal peristaltic pattern despite surgical trauma to the tissue. 8:00 am – 24 hour motility, 41 hours after sigmoid resection.

**Figure 4:**
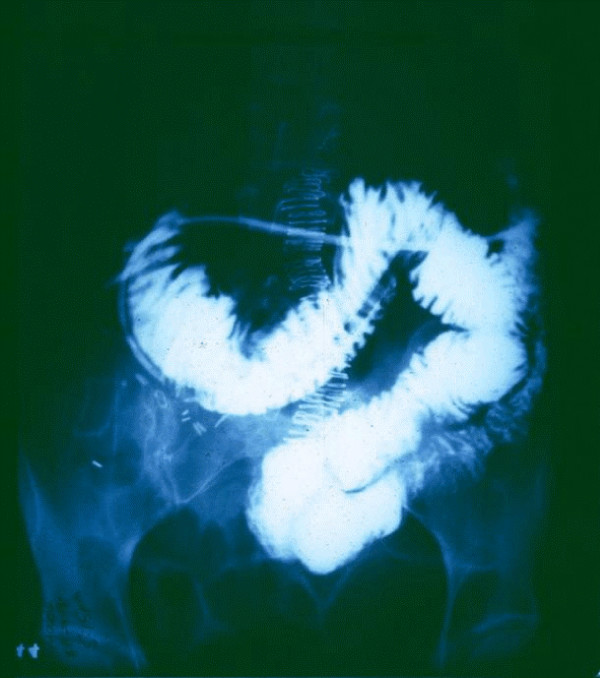
**54 y.o. woman had a right hemicolectomy for carcinoma @ 11:00 am**. Immediately fed with double lumen, feeding-decompression G-tube @ 300 kcal/hr. BaSO4 instilled via the feeding channel at rate exceeding peristaltic outflow. Note that no refluxing BaSO4 escaped aspiration, to enter the stomach. 4:00 pm – 5 minute motility, 5 hours after surgery.

**Figure 5:**
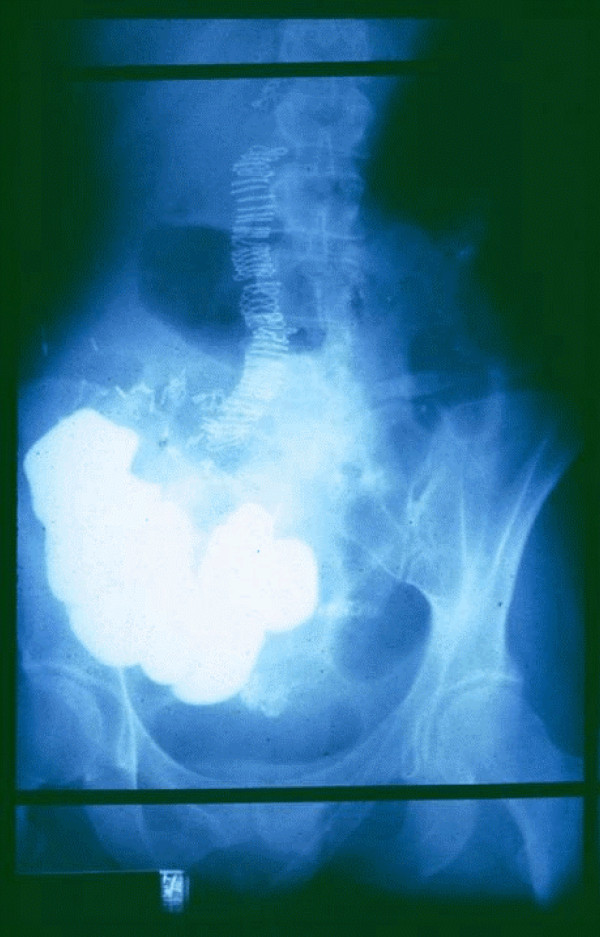
**Clinically normal motility**. Contrast is in the distal small intestine. 8:00 pm – 4 hour motility, 9 hours after a right hemicolectomy.

**Figure 6:**
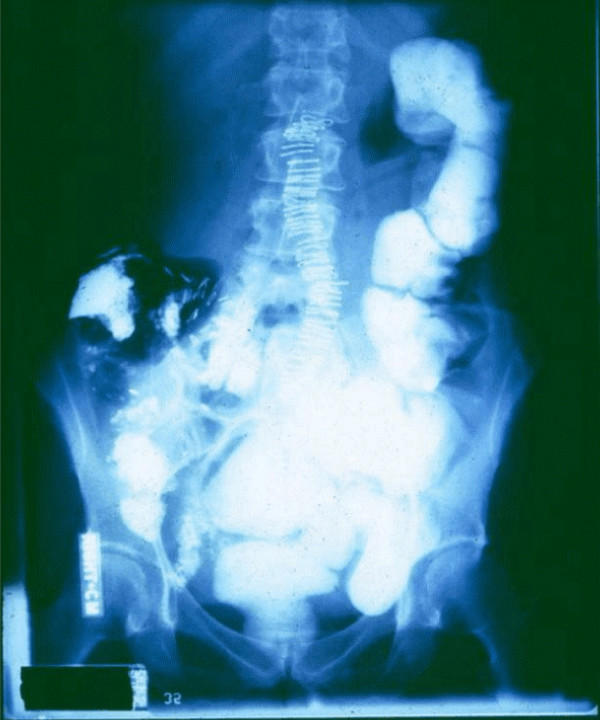
**Normal motility**. Dilute BaSO4 in the the colon and rectum. She tolerated a general diet, had a bowel movement, and was discharged within 24 hours of resective surgery. Note the laxative action of the excess feedings. 8:00 am – 16 hour motility, 21 hours after a right hemicolectomy.

X-ray motility studies showed clinically normal peristalsis, with contrast traversing secure, patent, and functional anastomosis, to outline the rectum within 24 hours. Permission was obtained to publish the studies. (Figures 1, 2, 3, 4, 5 and 6).

The median time to discharge decreased to 48 hours, with the final four colectomy patients home uneventfully 24 hours after bowel resection and re-anastomosis. The 160:160 consecutive "open" cholecystectomy patients were discharged within 24 hours of surgery.

## Discussion

Wangensteen studied the factors affecting gastrointestinal function and dysfunction. He reported 70 years ago that even the disruptive consequences of complete small bowel obstruction could be aborted if swallowed air was completely excluded [4]. He over-sewed the dog's terminal ileum, and simultaneously vented its cervical esophagus. His experimental subjects were adequately hydrated by "clysis," but were not otherwise nourished. They survived without intestinal distention for up to two months, ultimately dying of starvation.

**Figure 7:**
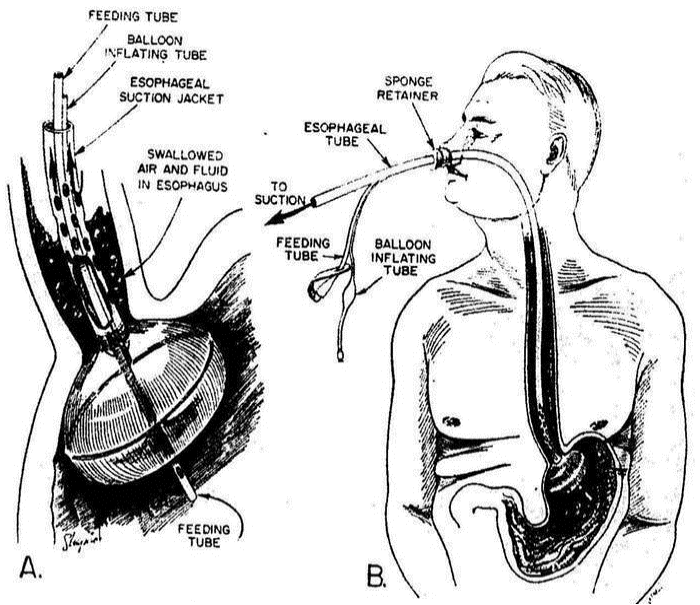
**Progenitor Moss7 Nasal Tube (circa 1961) Reprinted with permission of the American College of Surgeons**. Moss G. Nitrogen equilibrium in the early postoperative period. *Surg Forum *1963; 14:67–69.

We developed a labor intensive feeding-decompression regimen that mimicked Wangensteen's animal model, which we utilized experimentally and clinically for immediately postoperative gastric feeding [5] (Figure 7). In 1963 we reported on our first 24 patients at the Surgical Forum of the annual American College of Surgeons meeting [6].

The duration of clinically significant paralytic ileus was abbreviated to within two hours postoperatively. Positive protein balances similarly were achieved within hours of surgery. Nutrient absorption and X-ray motility studies documented the more rapid return of normal G-I function. To this day, that regimen allowed the earliest achievement of positive protein balances following colectomy or other trauma.

The patient's esophagus was aspirated to remove all swallowed air. Undiluted tracheo-bronchial secretions also were intercepted, demanding continuous irrigation for patency of the suction channel. Unlike Wangensteen's animal model, we provided a separate gastric channel for feeding and manually return of the filtered aspirate. Fear of gastric dilatation further increased the nursing workload, as we were obligated to frequently "check for residual."

The process was labor intensive, messy, and seldom performed outside of the research environment. The need for herculean nursing attention precluded wider acceptance of the original regimen. None-the-less, our clinical efforts were rewarded with preserved immediately postoperative G-I function.

Kehlet's Danish team reports on "fast-track" laparoscopic colectomy [7,8]. They conclude that application of advanced techniques ".... decreased the duration of ileus after colonic surgery to about 2 days, as compared with the usual 3 to 5 days." In Germany, Schwenk, et al., found the time to both bowel movement and tolerance of oral feeding after laparoscopic colo-rectal resection is shortened significantly, but still approximates three days [9]. Hammarqvist, et al., report patients undergoing laparoscopic cholecystectomy develop days of negative protein balance and loss of muscle mass, despite receiving total parenteral nutrition [10].

**Figure 8:**
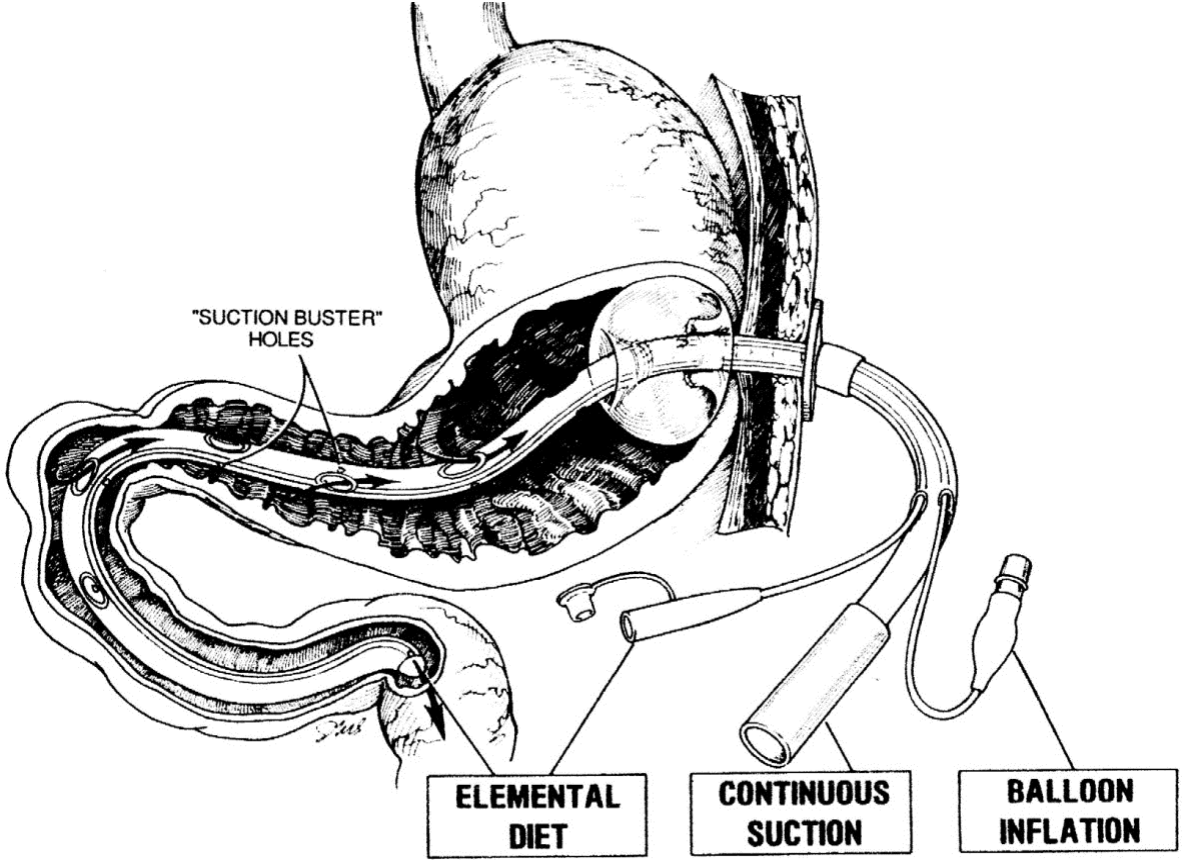
**Current Moss Gastrostomy Tube (circa 1985)**.

The major shortcomings of our earliest catheters had been frequent clogging of the esophageal aspiration lumen. As modified since 1985, aspiration was extended distally into the proximal duodenum [11] (Figure 8). Air and liquid were intercepted and removed efficiently within the close confines at this location, confirmed radiographically. A separate duodenal channel fed elemental diet (plus the returned, "degassed" aspirate) just distal to the aspiration orifices.

We reported that decompression efficiency with this approach is more than twelvefold greater than gastric aspiration alone by conventional nasal or gastrostomy tube [12]. Suction applied to the stomach can be expected only to prevent gastric dilatation. The post-pyloric aspiration orifices remove air and digestive secretions that escape proximal removal, efficiently decompressing within this narrow segment of duodenum.

There is scant duodenal resistance to retrograde flow of excess feedings that outstrip peristaltic outflow. These reflux and also are removed by efficient suction within these close confines, apparently before vagal reflexes can be induced. No liquid or gas can traverse this narrow segment in either direction without being intercepted (Figure 4).

Increased volumes of aspirated digestive juices are removed to dilute the swallowed phlegm, and also to remove excess feeding. This "trade-off" to reduce the nursing workload substitutes the equivalent of a high output duodenal fistula, aspiration of 3,000 – 4,000 ml per day of gastric and pancreatic juices, saliva, bile, and succus. Without "refeeding," the patient requires additional intravenous infusions to replace this fluid, as well as laboratory studies to guide restoration of electrolyte balance.

Aspirate became devoid of elemental diet within two hours of surgery, using Clinatest^® ^tablets as a check for the contained carbohydrate. In my personal experience with hundreds of surgical patients immediately fed @ 100–200 kcal/hour, elemental diet spontaneously propelled by peristalsis beyond the ligament of Treitz continues prograde, where it is totally absorbed. We accidentally (and later deliberately) fed many patients at even higher rates. They suffered no ill effects when their capacity to absorb was exceeded. With air excluded, the unabsorbed elemental diet serves as a mild, self-limited cathartic (Figure 6) (Laxatives currently are an accepted component of "fast-track" regimens.). None of our patients manifested "feeding intolerance," with increasing distention, nausea, etc.

Absorption of a high carbohydrate load is analogous to superimposing a "glucose tolerance test" upon surgical stress, further increasing the patient's insulin requirement. Otherwise healthy young cholecystectomy patients (< 24 years of age) respond by spontaneously raising their serum insulin levels as much as ten-fold above basal [13]. Diabetics and older (> 35 years of age) patients require insulin supplementation for optimum metabolic balance to avoid hyperglycemia during the initial 24 hours postoperatively. The patient reverts to his preoperative insulin requirement thereafter. Close plasma glucose control for seriously stressed patients is becoming a common practice, and is advised with postoperative "enteral hyperalimentation."

Clinically normal G-I motility and absorption resumed within hours for our 31 "open" colectomy patients, confirmed by the contrast X-ray studies. They consistently entered positive protein balance after institution of full feeding [14]. Contrast traversed secure and functional anastomosis, to outline the rectum and exit spontaneously in a bowel movement. Their usual course was discharge after 24–48 hours of "open" bowel resection and re-anastomosis [15].

For our series of 160 consecutive "open" cholecystectomy patients before laparoscopy was introduced into general surgery, discharge uniformly was within 24 hours [16,17]. Empire (New York State) Blue Cross, jointly with the Visiting Nurse Association of Albany, prospectively studied 19 consecutive patients. They were interviewed preoperatively, the day of surgery, the day after discharge, and again 30 days later. All had continued adequate food intake and fully participated in "activities of normal living" [18].

Our digestive secretions contain large quantities of secretory globulins that are specific for our enteric organisms. Refeeding these secretions, and/or immediately heightened antibody synthesis, may provide sepsis protection. Our single infection (in an acute cholecystitis patient) following 160 consecutive operations, is an apparent 90% reduction in sepsis that supports this hypothesis.

Refeeding digestive secretions and immediate postoperative feeding may mimic prophylactic antibiotics, at lower financial and health costs. This requires further clinical study.

We consistently noted that elevation of serum amino acids within two hours accompanied positive protein balance and net protein synthesis [19]. Serum branched chain amino acids rose immediately, rather than falling below basal for several days. This was confirmed by a surgical team in Cleveland in 34 consecutive "open" cholecystectomy patients fed with our regimen. Branched chain amino acids, essential for protein preservation and synthesis, rose above basal within one hour, and was sustained for the duration of feeding [20].

Experimental wounds reportedly benefit from this procedure to safely initiate early nutrition. We applied this regimen to dogs subjected to bowel resection. By 72 hours postoperatively for fed versus control beagles, wound DNA synthesis was increased six-fold.

By 96 hours, large bowel, intestinal, and abdominal wall wounds had bursting strengths doubled, tripled, and quadrupled, respectively. The stronger wounds showed accelerated collagen synthesis, i.e., procollagen content had doubled. Mature wound collagen of control dogs had diminished 50%, while remaining unchanged for fed subjects [21]. A study of colectomy patients using our feeding-decompression regimen in New Zealand confirmed early increased wound procollagen in their experimental, simultaneous forearm wounds [22].

Immune protein synthesis shared in the accelerated protein synthesis associated with immediate "enteral hyperalimentation." Production of immune globulins doubled experimentally during the initial 24 hours [23]. Our animal findings were reproduced clinically in a controlled study of radical urological surgery at Roswell Park (Buffalo, NY) [24,25]. Their immediately fed patients achieved statistically significant higher levels for both plasma amino acids and immune protein (fibronectin) than unfed controls.

With this regimen, secretory globulins in digestive juices that target enteric organisms are neither removed nor sequestered, but carried to the patients' lower G-I tract soon after surgery. These may provide protection against nosocomial infection during the early postoperative period, when the patient is most vulnerable to infection by his own gut organisms.

The traumatized and vulnerable patient pays a hidden "price" for delayed or suboptimal enteral nutrition. Härtl, et al., reported a prospective study of 797 severe TBI (traumatic brain injury) patients treated at 22 New York State trauma centers from 2000–2006 [26].

"Patients who were not fed within 5 and 7 days after TBI had a 2- and 4-fold increased likelihood of death, respectively. The amount of nutrition in the first 5 days was related to death; every 10-kcal/kg decrease in caloric intake was associated with a 30–40% increase in mortality rates."

Jeschke, et al., showed that forced feeding lessened the hypermetabolic immune consequences in their scalded rats [27]. Unlike humans, rats have resilient G-I tracts that function well despite such insult. However, humans have a parallel immune response to hypermetabolic stresses and tolerable nutrition changes.

For our regimen, the feeding solution used must be of low viscosity, and remain completely soluble after contact with acidic or alkaline juices (e.g., casein denatures to becomes "cheese"). Amino acids substitute nutritionally for protein in "elemental diets." This is the only type of feeding solution that we have found consistently suitable for this regimen, totally avoiding potentially obstructing whole protein and fiber for the initial 24 hours [28].

We recently reported a regimen to automate outflow monitoring from the feeding site and "refeed" the aspirate after esophagectomy. The results were identical to our previous manual experience with postoperative immediate feeding, but with a reduced nursing workload [29].

"Feeding intolerance" becomes evident only after gut recovery already has been aborted. Because "clinical judgment" alone is unreliable and "overfeeding" so detrimental, clinicians at Memorial – Sloan Kettering Cancer Center (New York, NY) [30] and the University of Ottawa [31] concluded that attempts to enterally feed immediately postoperatively were not warranted.

The New York team prospectively studied 100 pairs of matched cancer patients after resective abdominal cancer surgery. Half received closely supervised early jejunal feeding. The fed patients recovered more poorly, including one that developed bowel necrosis. They concluded that immediate enteral feeding after major resective surgery does not warrant the risks.

The Ottawa team also found that their early postoperative enteral feeding impaired recovery. They monitored pre- and postoperative mobility and pulmonary mechanics, to gain insight into why feeding proved detrimental. All their postoperative patients initially were bedridden. However, their fed patients were more severely affected, exhibiting poorer respiratory function, increased abdominal distention, and greater immobility.

This feeding-decompression regimen safely permits the earliest enteral feeding, despite initially impaired G-I function. More rapid return and utilization of gut function appears to reduce sepsis, mortality, discomfort, and hospital length of stay. Determining optimum nutrition in the face of the heightened metabolic demands of trauma requires further controlled study, but the "feeding intolerance" of paralytic ileus safely can be removed as a confounding factor.

## Conclusion

The symptom complex of "feeding intolerance" most likely is triggered by localized distention at the enteral feeding site. It can be prevented by removing any excess liquid and all swallowed air from this location. A feeding-decompression regimen is reported that:

1. aspirates digestive juices and air from within the stomach and proximal duodenum;

2. delivers feeding solution into the slightly more distal duodenum;

3. removes excess feedings before localized distention can develop;

4. filters and returns the "degassed" aspirate via the feeding channel.

Automation can reduce the mess and nursing work-load of salvaging the aspirated digestive juices.

## Competing interests

The author declares that they have no competing interests.

## Disclaimer

Relating to p.5:

*For the past decade, Dr. Moss has had no financial affiliation with Moss Tubes, Inc., which manufactures Moss^® ^medical devices and owns the trademark.
